# CHANGES IN DRIVER’S LICENSE STATUS AMONG MID-AGED AND OLDER CANADIANS OVER THREE YEARS

**DOI:** 10.1093/geroni/igad104.2174

**Published:** 2023-12-21

**Authors:** Arne Stinchcombe, Shawna Hopper, Sylvain Gagnon, Michel Bedard

**Affiliations:** University of Ottawa, Ottawa, Ontario, Canada; Simon Fraser University, Vancouver, British Columbia, Canada; University of Ottawa, Ottawa, Ontario, Canada; Lakehead University, Thunder Bay, Ontario, Canada

## Abstract

For older people, driving may contribute to health and quality of life. Conversely, driving cessation is associated with negative outcomes, including poor physical and mental health. We examined changes in driving status over a three-year period among participants in the Canadian Longitudinal Study on Aging (CLSA), which includes Canadians aged 45-85 at baseline. At baseline (data collected between 2011 and 2015) and follow-up (data collected between 2015 and 2018), participants reported whether they had a driver’s license. Using multiple logistic regression, we examined the relationship between covariates and changes in license status (i.e., having a license at baseline but not at follow-up vs. maintaining license). Of the participants who reported having a driver’s license at baseline (n=36,266), 1.19% (n=432) reported no longer having one at follow-up. This change was associated with lower income categories and poorer self-rated health. Age (B=.12, p<.001) and depression symptoms (B =.04, p<.001) were positively associated with no longer having a license. Participants who reported a change had lower scores on a memory task (Rey Auditory Verbal Learning Test; B=-.07, p<.001). Women had greater odds than men to report a change in driver’s license at follow-up (OR=1.5, p<.001). The results highlight the salience of health, cognition, and income as correlates of driving cessation in a sample of mid-aged and older adults. These results may help identify individuals who are likely to stop driving and who may need additional supports maintaining mobility, health, and quality of life.

